# The psychological and behavioral effects of social isolation on mass shooters

**DOI:** 10.3389/fpsyt.2025.1686510

**Published:** 2025-10-21

**Authors:** Adam Lankford, Jason R. Silva

**Affiliations:** ^1^ Department of Criminology and Criminal Justice, The University of Alabama, Tuscaloosa, AL, United States; ^2^ Department of Sociology and Criminal Justice, William Paterson University, Wayne, NJ, United States

**Keywords:** mass shooting, mass shooters, social isolation, mental health, violence prevention

## Abstract

**Background:**

Social isolation has been identified as a risk factor in the lives of mass shooters, assassins, serial killers, child molesters, lone actor terrorists, suicide attackers, and other violent offenders, but its psychological and behavioral effects are only partially understood.

**Methods:**

For this article, we tested for bivariate differences between public mass shooters who were and were not socially isolated in the United States from 2000 to 2024.

**Results:**

Compared to other perpetrators, socially isolated mass shooters were significantly more likely to be unemployed, single, childless, and sexually frustrated; to have a mental health problem in general, autism specifically, prior psychiatric hospitalization, and a history of suicidality (unrelated to their attack intentions); to use substances, play violent video games, adopt prejudices, seek fame, and show interest in past mass violence; and to kill more victims.

**Discussion:**

Based on these findings and other research, we propose a new model for social isolation’s effects on mass shooters’ mental health and their coping mechanisms prior to attack. We also provide illustrative case examples and offer recommendations for future research.

## Introduction

Socially isolated individuals—or “loners”—have historically been regarded with suspicion (1–4). Perhaps this is because humans are hardwired to be social animals and interact in a variety of mutually beneficial ways (5–7). A recent report by the U.S. Surgeon General entitled “Our Epidemic of Loneliness and Isolation” (8) asserts that “Social connection is a fundamental human need, as essential to survival as food, water, and shelter” (p. 9). Asocial or anti-social preferences might therefore be assumed to be unnatural or unhealthy.

If someone is a loner by choice, that could raise questions about their desire for seclusion and whether they have something to hide; if someone is a loner against their will, they have typically been ostracized due to some perceived offense. Accordingly, social isolation, which the U.S. Surgeon General (8, p. 7) defines as “Objectively having few social relationships, social roles, group memberships, and infrequent social interaction,” has been identified as a risk factor in the lives of mass shooters, assassins, serial killers, child molesters, lone actor terrorists, suicide attackers, and more (1–3, 9–17).

However, we believe a great deal remains to be learned about the psychological and behavioral effects of social isolation on violent offenders. For instance, much research suggests that social isolation can have damaging consequences on people’s mental health (8). Although such isolation may not be the sole cause of mental health problems, it could be one of many factors that triggers or exacerbates symptoms of psychiatric disorders (e.g., depression, paranoia, psychosis, anxiety disorders, autism spectrum disorder) or renders coping mechanisms ineffective (18–21). As a result, socially isolated individuals with a given disorder may suffer more than socially connected people with the same psychological condition. This could help explain why the interactions between mental health, self-harm, and violence are so complex and in need of further elucidation (22, 23), but current knowledge is primarily derived from studies of the general population, not violent offenders (8, 18–21).

Similarly, research suggests that in general, social isolation may increase individuals’ use of social media, online chat groups, and violent video games (24–26), but that evidence does not come from studies of violent offenders. If violent offenders do respond to social isolation in these ways, that could help explain their adoption of extremist or prejudiced attitudes, their interest in violent role models, or their desire to achieve fame or social recognition at any cost (12). In turn, such beliefs, attitudes, and motives may drive their decision to harm others (2, 13, 17, 27, 28).

As a starting point, however, it would help to know more about the relationship between social isolation and other psychological and behavioral factors among a sample of violent offenders, such as public mass shooters. For example, although previous studies have documented that public mass shooters are often socially isolated, for the most part they have not tested for associations with other factors in perpetrator’s lives (11, 12, 16, 17, 29). A rare exception is a recent study by West and Thomson (30), which found that isolation may exacerbate other issues, such as mass shooters’ difficulty with daily tasks.

More broadly, if social isolation is closely associated with mass shooters’ mental health problems and pre-attack behaviors, that could have important theoretical and practical implications. For example, in accordance with general strain theory (31), that could indicate that perpetrators often experience interconnected strains which lead to violence; while from the perspective of social control theory (32), it could indicate that having stronger social bonds would function as a protective factor to reduce the risks. From a practical perspective, if social isolation is associated with mental health problems and potentially dangerous behaviors, that could inform threat assessment and management strategies for violence prevention.

## Method

For this article, we tested for bivariate differences between public mass shooters who were and were not socially isolated. We analyzed data on all public mass shooters (*N* = 123) who attacked in the United States from 2000 to 2024 according to The Violence Project (TVP) database, version 9, which was funded by the National Institute of Justice and compiled by Peterson and Densley (33). Their definition of public mass shootings is drawn from the Congressional Research Service:

  Incident[s] in which four or more victims are murdered with firearms—not including the offender(s)—within one event, and at least some of the murders occurred in a public location or locations in close geographical proximity (e.g., a workplace, school, restaurant, or other public settings), and the murders are not attributable to any other underlying criminal activity or commonplace circumstance (armed robbery, criminal competition, insurance fraud, argument, or romantic triangle) (34, p. 10).

Most data came from TVP, which is open access for interested readers. TVP’s coding of social isolation is consistent with the aforementioned definition from the U.S. Surgeon General (8), with particular consideration of perpetrators experiencing a recent decline in social connections, which may be especially relevant to attack-related psychology and behavior. We also examined many other variables, including mass shooters’ age, sex, employment status, relationship status, parental status, sexual frustration, mental health problems, autism spectrum disorder, childhood trauma, psychiatric hospitalization, suicidality, substance use, violent video game use, hate group/chat room affiliation, extreme ideological interests, known prejudices, fame-seeking, interest in past mass violence, and how many victims they killed and injured. Within the exception of age and victims killed/injured, which are continuous measures, the other variables were all coded as 1 = yes, 0 = no/no evidence for this study. The measure for mental health problems includes both individuals who were formally diagnosed with psychiatric disorders and individuals who displayed clear symptoms of mental illness despite a lack of a documented direct assessment. Our childhood trauma variable included physical, sexual, and emotional abuse and death of a parent during childhood. We included the five items on mental health problems, autism spectrum disorder, childhood trauma, psychiatric hospitalization, and suicidality because they provide a multifaceted overview of each individual’s mental state and were available in TVP’s database. Beyond autism spectrum disorder, TVP does not contain information on most other specific disorders (e.g., generalized anxiety disorder, obsessive-compulsive disorder), so including them was not an option. We included the items on substance use, violent video game use, hate group/chat room affiliation, extreme ideological interests, known prejudices, fame-seeking, and interest in past mass violence because they may be attractive to socially isolated individuals seeking to dull their pain, entertain themselves, blame others, or become notorious—and because those data were also available. We used the aforementioned data from TVP, updating any missing information, and supplemented it with findings from other research (27, 28, 35, 36).

## Results

As shown in **Table 1**, our quantitative comparison of public mass shooters with and without a history of social isolation revealed many significant differences. Bivariate tests showed that socially isolated offenders were more often unemployed, single, childless, and sexually frustrated than socially connected offenders. They were also significantly more likely to have a mental health problem in general, autism specifically, prior psychiatric hospitalization, and a prior history of suicidality (that was unrelated to their attack intentions). Socially isolated mass shooters were also more likely to use substances (drugs, alcohol), play violent video games, adopt prejudicial attitudes, seek fame and attention, and show interest in past mass violence. Regarding their attacks, socially isolated mass shooters killed significantly more victims than other mass shooters.

**Table 1:** Comparing public mass shooters with and without a history of social isolation.

Variables	Socially isolated (*n* = 56)		Not Socially isolated (*n* = 67)			
	*n*	*%/Avg.*	*n*	*%/Avg.*	*X^2^/T-test*	*p-value*
*Profiles*						
Age	–	33.48	–	34.43	-0.412	
Sex (Male)	54	96.4%	64	95.5%	0.064	
Unemployed	42	75.0%	39	58.2%	3.825	*
Single	49	87.5%	36	53.7%	16.293	***
Childless	47	83.9%	42	62.7%	6.882	**
Sexually Frustrated	26	46.4%	17	25.4%	5.948	*
*Mental Health*						
Mental Health Problems	52	92.9%	40	59.7%	17.788	***
Autism Spectrum Disorder	9	16.1%	2	3.0%	6.739	**
Childhood Trauma	16	28.6%	12	17.9%	1.972	
Prior Psychiatric Hospitalization	17	30.4%	10	14.9%	4.240	*
Prior History of Suicidality (Unrelated to Attack Intent)	27	48.2%	16	23.9%	7.944	**
*Potential Coping Mechanisms*						
Substance Use	28	50.0%	20	29.9%	5.205	*
Violent Video Game Use	25	44.6%	15	22.4%	6.885	**
Hate Group/Chat Room Affiliation	17	30.4%	11	16.4%	3.371	
Extreme Ideological Interests	19	33.9%	17	25.4%	1.079	
Prejudices	28	50.0%	19	28.4%	6.051	**
Fame-Seeking	21	37.5%	11	16.4%	7.044	**
Interest in Past Mass Violence	28	50.0%	17	25.4%	7.974	**
*Attack Consequences*						
Victims Killed	–	9.50	–	6.48	2.113	*
Victims Injured	–	6.55	–	5.39	0.650	

*N* = 123; *p <.05. **p <.01. ***p <.001. Injuries were calculated without 2017 Las Vegas outlier.

## Discussion

Overall, our findings suggest that socially isolated public mass shooters tend to suffer more than other mass shooters in terms of their mental health and life circumstances, are more likely to engage in certain unhealthy behaviors, and are prone to commit more destructive attacks. Statistically, we could not test for causation—only associations—but we believe some important causal pathways exist.

**Figure 1:**
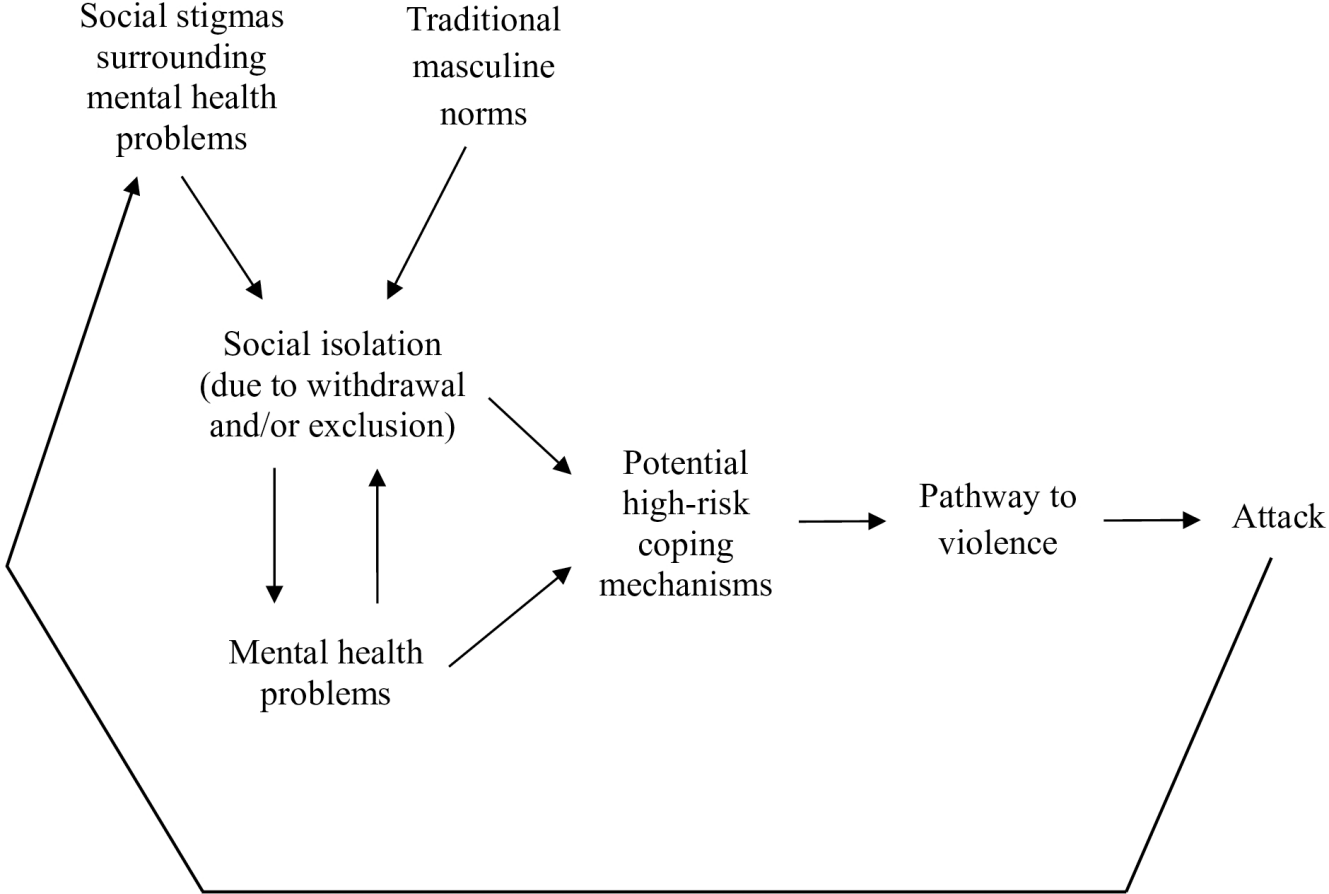
Proposed model of social isolation's effect on mass shooters.

In **Figure 1**, we propose a new model to explain social isolation’s effects on public mass shooters that we describe in more detail below. This model is informed by both our quantitative findings on this subject and our perspective from many years of studying mass shooters. However, it is still conceptual at this point and would benefit from direct testing.

- First, social isolation and mental health problems can affect each other. Social isolation can trigger or exacerbate symptoms of psychiatric disorders (e.g., depression, paranoia, psychosis, anxiety disorders, autism spectrum disorder) (18–21). In addition, people with mental health problems may socially withdraw or be socially excluded by those around them (37, 38). Some disorders clearly inhibit people’s ability to socialize effectively, which is a big part of the challenge (8, 19, 20).
- Second, these individuals’ social isolation is also affected by the cultural context. For instance, social stigmas surrounding mental illness that depict it as a threat, and traditional masculine norms that teach men to hide their pain, may both increase people’s likelihood of social isolation (22, 39, 40). Some may feel ashamed and attempt to conceal their own struggles by socially withdrawing, while others may be ostracized due to discriminatory attitudes.
- Third, given the importance of social connection for nearly all human beings (5–8), socially isolated individuals need ways to cope. Some potential coping mechanisms are healthy (e.g., asking for help, seeking treatment, finding new hobbies) but others come with higher risks, like substance use, violent video games, prejudices, fame-seeking, and interest in past mass violence. We describe these as potential coping mechanisms because they could (a) provide distraction or entertainment, (b) offer opportunities for social connection, (c) help the individual join an “ingroup” that is united against an “outgroup,” or (d) feed the individual’s fantasies of becoming socially popular within a fringe community. Increasingly, people who are socially isolated in the physical world seek social connection online, but that can increase their risks of radicalization (12) and does not yield equivalent mental health benefits as face-to-face friendships (41–43).
- Fourth, in cases where high-risk coping mechanisms fail, individuals may be more likely to proceed on a pathway to violence. This pathway has historically been conceived of as a series of escalating and increasingly dangerous steps that individuals take when they intend to commit targeted violence (44). For instance, their grievances can lead to violent ideation, which can lead to research and planning, followed by preparation, breach, and attack (45, 46). As an example of how this would fit with our model, those individuals who feel socially excluded and have adopted prejudices or interest in past mass violence may be more prone to develop targeted grievances and believe violent retribution is justified. That could start them on a pathway to violence that ends in tragedy.
- Finally, if these individuals commit a public mass shooting, the news of their attack and their psychiatric background can then feed greater social stigmas about mental illness. This increases the likelihood that people who are struggling in the future will become socially isolated—and so the cycle continues.

Of course, this model does not include descriptions of all offenders, and other variations certainly exist. However, we are familiar with many case examples of public mass shooters who appeared to follow this trajectory, in part or in full. For example, the 2011 Tucson shooter was diagnosed with schizophrenia and posted that participating in online forums “is like my social life” (47). He was suspended from college—which only increased his isolation—used illegal drugs, struggled with sexual frustration, and sought fame for his attack. Similarly, the 2015 Charleston Church shooter was diagnosed with social anxiety disorder, a schizoid personality disorder, depression, and a possible autistic spectrum disorder (48). He complained online that “My life is wasted. I have no friends even though I am cool,” used illegal drugs, and developed racial and anti-Semitic prejudices prior to his attack (47). As a final example, the 2018 Walmart shooter struggled with mental health problems since childhood, was diagnosed with schizoaffective disorder with symptoms that included hearing voices and difficulty processing feelings, and was described as “very much a loner, very standoffish” (47, 49). He started playing violent video games, visited online hate forums, adopted prejudiced and extremist views, sought fame and attention, and developed an interest in past mass violence (12). For each of these mass shooters and many others, there were complex interactions between their social isolation, mental health problems, coping mechanisms, and violent attacks.

## Conclusion

The ideas and data we have offered here are only the first steps. Much more can still be explored. For instance, research on non-human animals has found causal links between social isolation and subsequent aggressiveness, which suggests a biological mechanism that may be applicable to humans (56, 50). There is also a long human history of banishment and solitary confinement being used as punishments (51), even though the National Academies of Science, American Psychological Association, and National Commission on Correctional Health have warned about damaging effects on mental health (52). This raises questions about whether, at some level, mass shooters who are socially isolated against their will (e.g., excluded by peers, suspended or expelled from school, fired or laid off from work, dumped by an intimate partner) feel unfairly punished—which could motivate their decision to attack. Social isolation may also be an important factor in hostile attribution bias (53)—the dangerous tendency some individuals (including many mass shooters) have of interpreting others’ intent as hostile, even when it actually appears benign (54, 55).

From a more optimistic perspective, however, there is a large body of research that suggests building social connections can have strong protective effects (8, 15, 19). This is possible for nearly everyone, even if they are living with mental health problems. Along with each individual’s efforts to increase meaningful social connections in their own lives, there are many groups that can help, including government organizations, community-based organizations, media and entertainment corporations, technology companies, public health professionals, schools, workplaces, and families (8). We sincerely hope these avenues for threat management and violence prevention are more fully investigated by researchers, practitioners, and community members alike.

## Data Availability

Publicly available datasets were analyzed in this study. This data can be found here: https://www.hamline.edu/violence-prevention-project-research-center.
